# The climate crisis is a global health emergency: A call to arms

**DOI:** 10.1016/j.fhj.2025.100238

**Published:** 2025-03-31

**Authors:** Matthew RD Lee, Mark Harber

**Affiliations:** a Climate Change and Healthcare Sustainability to the Royal College of Physicians, London, UK; b Doctors’ Association, London, UK; c Cardiff University, London, UK; d Department of Renal Medicine, UCL, London, UK

The year 2050 is often referenced as a future milestone in climate change conversations. Governments discuss how decarbonised they want the country by this time, researchers talk about expected sea level rise and anticipated number of refugees or severity of extreme weather events by this time, often with staggeringly bleak statistics.

However, 2050 is much closer than we like to think. To put that year into the context of training a doctor, thousands of current resident doctors will still be over a decade away from retirement in 2050. Those entering their first year of medical school this September will be consultants. A ‘future doctor’ born today will likely be in their foundation years, or even just starting specialty training. Perhaps more disconcertingly for some of the current consultant readership, the year 2050 might be the first time they see the other side of an older persons’ ward!

The sixth assessment report by the Intergovernmental Panel on Climate Change (IPCC) summarised the urgent timeframe by stating that ‘Climate change is real, man-made, rapid, and unprecedented. Temperatures will continue to rise under all scenarios. Species extinction, widespread disease, unliveable heat, ecosystem collapse, and cities menaced by rising seas will become painfully obvious before a child born today turns 30’.^1^

This editorial aims to illustrate the health impacts of the climate crisis and encourage physicians to be at the leading edge of change. Murray and Montgomery^2^ lay out the stark state of the planet now, the events to come and impacts already here. This is not just speculation, this is based on the work of tens of thousands of scientists who have been accurately predicting the impacts of burning fossil fuels, for decades.

Climate change is already having very direct effects on health in terms of the epidemiology of arthropod-borne vectors presenting physicians and public health teams with new and emerging infectious diseases.^3^ In addition, Maslin *et al*^4^ and Dey^5^ both describe the non-communicable health effects of climate change. This has profound implications for healthcare with an increasingly comorbid and elderly population.

Montero^6^ describes the practical risks of climate change on provision of healthcare from extreme weather events to supply chain shocks and the resilience, or otherwise, of the health service in delivering time-dependent care. Allied to this is the fact that displacement of peoples internally and across borders on a massive scale is an inevitable consequence of increasing drought, famine and flooding. Providing care for such populations is a challenge both for the global community and the medical profession that needs a strategy starting immediately.

Kassam and Smith^7^ explore how our dietary habits affect our health, presenting a strong case for clinicians to engage directly on discussions with patients on shifting to more sustainable, plant-based diets. These authors provide recommendations for healthcare leaders to support such a transition, along with strong evidence on the negative health impacts that our current diets are causing.

As physicians, we must understand that climate change is a health and humanitarian crisis that will impact most heavily on the poor and disposed, but very rapidly on all of us on a scale never seen before. The ‘business as usual’ approach that has so characterised so much of global governmental, industrial and institutional response to the ‘inconvenience’ of climate crisis over the last few decades is responsible for terrible harm. The article by Murray and Montgomery gives many practical steps that we as individuals can take, and other articles in this edition recommend things that we can do in our departments and medical communities. For the sake of our patients, healthcare professionals must be at the forefront of tackling climate change, demonstrating leadership through radical, systemic climate action to accelerate the decarbonisation process of the NHS, the wider UK, and influence a global transition to a safer, liveable future.

## Funding

This research did not receive any specific grant from funding agencies in the public, commercial, or not-for-profit sectors.

## Declaration of competing interests

Matthew RD Lee is the deputy special adviser on climate change and healthcare sustainability to the Royal College of Physicians (RCP). He is the sustainability lead for Doctors’ Association UK and an honorary lecturer for Cardiff University. Mark Harber is special adviser on climate change and healthcare sustainability to the RCP.
